# Romosozumab and Denosumab Combination Therapy After Denosumab in Postmenopausal Osteoporosis

**DOI:** 10.1002/art.70002

**Published:** 2026-02-22

**Authors:** Giovanni Adami, Francesco Pollastri, Angelo Fassio, Filippo Montanari, Anna Piccinelli, Camilla Benini, Emma Pasetto, Carmen Dartizio, Davide Gatti, Maurizio Rossini, Ombretta Viapiana

**Affiliations:** ^1^ Rheumatology Unit University of Verona Verona Italy

## Abstract

**Objective:**

Transition from long‐term denosumab (Dmab) to parathyroid hormone‐analogs or romosozumab (Romo) might expose patients to the risk of the so‐called rebound phenomenon. Adding Romo to Dmab might represent an option in patients experiencing a fracture while on Dmab. The aim of this study was to investigate the effects of the combination of Romo to Dmab in postmenopausal osteoporosis.

**Methods:**

We did a 36‐month combined retrospective and prospective study analyzed with prospective score matching. Postmenopausal women were divided into two groups: patients on Dmab who added Romo to Dmab (Dmab from baseline [M−24] to Romo initiation [M0] ➔ Dmab + Romo from M0 to M+12), and matched controls on Dmab continuing Dmab (Dmab from M−24 to M0 ➔ Dmab from M0 to M+12). Bone mineral density and bone turnover markers (CTX, P1nP) were assessed at follow‐up time points.

**Results:**

A total of 50 women were included in the study: 25 patients in the Dmab ➔ Dmab + Romo group and 25 matched controls in the Dmab ➔ Dmab group. The between‐group difference at M+12 was 3.3% (95% confidence interval −5.2 to 11.8), indicating a nonsignificant trend toward greater improvement with combination therapy. Adding Romo to Dmab increased P1nP significantly between M0 and M+3 (+22.5 ng/mL, SE = 8.7; *P* = 0.028).

**Conclusion:**

Ongoing treatment with Dmab did not blunt the anabolic response of Romo, indicating sustained modeling‐based bone formation activity. Adding Romo in patients failing Dmab might be a valuable option.

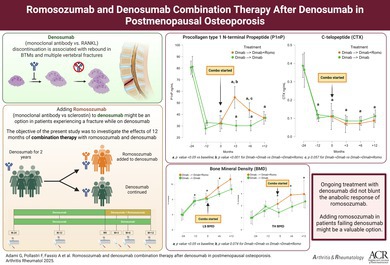

## Graphical abstract

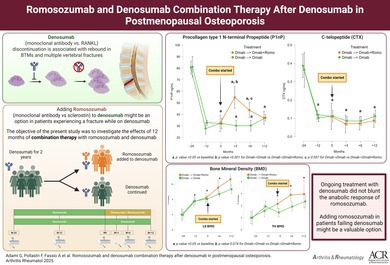

## INTRODUCTION

Osteoporosis is characterized by decreased bone mass and deterioration of bone microarchitecture, with increased fracture risk. Current approved treatments for postmenopausal osteoporosis include antiresorptive drugs (bisphosphonates and Dmab), anabolic agents (teriparatide and abaloparatide), and romosozumab (Romo), an antisclerostin monoclonal antibody with dual action on bone formation and resorption. Osteoporosis treatment regimens often include sequential therapies involving drugs with complementary mechanisms, and the sequential approach with anabolic first has been proved to be the most effective on bone mineral density (BMD) changes and fracture risk reduction.^1^ However, in real life, most patients start with an antiresorptive agent as first line. Switching from bisphosphonates to an anabolic agent is considered safe and effective, albeit with attenuated effects on BMD. However, transitioning from denosumab (Dmab) to teriparatide or Romo may expose patients to increased fracture risk due to the simultaneous activation of multiple bone remodeling units upon treatment cessation, known as the “rebound effect.” In such patients, anabolic initiation is usually prompted by treatment failure, defined as significant bone density loss and/or fragility fractures. Sequential therapy with Romo has also shown variable outcomes.^2^ Romo after long‐term alendronate led to lower BMD gains than observed in treatment‐naive patients. Switching from long‐term Dmab to Romo yielded only minimal BMD improvements and did not protect against multiple vertebral fractures upon Dmab discontinuation.^3^, ^4^ Our preliminary study demonstrated that adding Romo to ongoing Dmab treatment improved lumbar spine BMD significantly in six months.^5^ Moreover, we previously showed that Dmab increased circulating sclerostin levels,^6^ supporting the hypothesis that adding Romo could exploit this mechanism to enhance BMD responses in patients already on Dmab. In the present study, we aimed to evaluate the effects of 12 months of combination therapy with Romo and Dmab.

## MATERIAL AND METHODS

**Figure 1:**
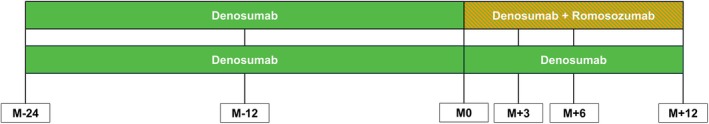
Study design. M, month.

We designed a combined retrospective and prospective 36‐month study analyzed with prospective score matching to evaluate the effects of Romo and Dmab combination therapy on postmenopausal women with severe osteoporosis. We did a 12‐month prospective observational study starting at month 0 (M0) (Romo initiation), complemented by retrospective data collection at 24 months before initiation (M−24) and M−12 from the patients’ medical records. The study design can therefore be described as a hybrid observational study, with retrospective and prospective components. Figure 1 depicts the study design. If a patient refused or was not eligible to be treated with combination, she was still considered for the Dmab‐only group and treated according to clinical practice. The study population was divided into two cohorts based on treatment exposure: (1) combination group, in which patients were treated with Dmab for two years, at which point Romo was added to ongoing Dmab: Dmab (M−24 to M0) ➔ Dmab + Romo (M0 to M+12), and (2) Dmab‐only group: patients who were stable on Dmab, and for whom continuing Dmab treatment alone was deemed appropriate by the treating physician: Dmab (M−24 to M0). Patients in the Dmab + Romo group were prospectively enrolled at M0 (when Romo was added to ongoing Dmab therapy). Study protocol is given in the Supplementary Materials. Enrollment occurred between December 2022 and January 2024. For these patients, we also retrieved retrospective data (M−24 to M0) from their charts to ensure longitudinal comparability. Patients were instructed to receive Dmab (60 mg subcutaneously) first, followed by Romo (210 mg subcutaneously) within seven days. This sequence was chosen to maintain the antiresorptive effect of Dmab while initiating the anabolic action of Romo without delay. All administrations were verified by patient report.

Patients in the second group (Dmab only) were selected from the overall pool of postmenopausal women treated with Dmab referring to our outpatient clinic (between January 2016 and March 2024) who had available baseline (M−24) and follow‐up dual‐energy x‐ray absorptiometry (DXA) scans and serum samples collected (n = 388). Patients were then matched through propensity score matching to balance baseline characteristics between the treated and control groups. The propensity scores were estimated using a logistic regression model. The following baseline covariates, selected based on clinical relevance and prior literature, were included in the model: age, sex, T score at femoral neck, total hip and lumbar spine, prior vertebral and nonvertebral fractures, and procollagen I intact N‐terminal peptide (P1nP) and C‐terminal telopeptide of type I collagen (CTX) serum levels. Patients were matched 1:1 using nearest‐neighbor matching without replacement, with a caliper width of 0.2 SDs of the logit of the propensity score to ensure close matches. Matching was performed at M0.

### Inclusion and exclusion criteria for the combination treatment (M0 to M+12)

The inclusion criteria were as follows: postmenopausal women with severe osteoporosis, defined as 10‐year major osteoporotic fracture risk ≥20%, assessed using the deDeFRA tool (a validated fracture risk assessment tool derived from FRAX)^7^, ^8^, ^9^ and T score at the spine or femur of less than −2.5 (or less than −2.0 if there were two or more moderate or severe vertebral fractures or a femoral fracture in the previous two years).

The exclusion criteria were as follows: history of myocardial infarction or stroke, bone diseases other than osteoporosis (eg, Paget disease), history of bone malignancy, severe liver or kidney disease (estimated glomerular filtration rate [eGFR] <30 mL/min or Child‐Pugh grade B/C), uncontrolled endocrine disorders (eg, hypocalcemia, primary hyperparathyroidism), treatment with bisphosphonates for more than 6 months in the 12 months preceding Dmab initiation, and prior or active treatment with glucocorticoids or hormone blocking therapy.

### Data collection

BMD measurements were taken at baseline (M−24) and at M−12, M0, M+6 and M+12 of therapy, at the femoral neck, total hip, and lumbar spine (L1–L4) using DXA with the QDR Hologic Delphi machine. The variation coefficient for the vertebral site was 1%, whereas it was 1.2% for the femoral neck. Blood samples were collected in the morning after fasting at M0, M+3, M+6, and M+12. The serum samples were aliquoted and stored at −80°C until they were assayed for bone turnover markers (BTMs): CTX (a marker of bone resorption) and P1nP (a marker of bone formation)

To provide a comprehensive analysis of the BTMs trends, we also collected M−24 and M−12 data retrieving the information from electronic medical records (M−24 to M0). These included CTX and P1nP, collected at M−24 and M−12 (ie, 24 and 12 months before M0, ± 1 month). This approach allows for a longitudinal view of BTMs and BMD both before and after the initiation of Romo or continuation of Dmab, enabling the comparison of treatment effects over time. All participants received calcium and vitamin D according to clinical practice (>1,000 IU/day of vitamin D) +1,200 mg/day of calcium (dietary + supplements combined if needed).

### Sample size considerations

We previously observed that the difference in lumbar spine BMD between combination of Romo and Dmab versus Dmab alone was around 6% at lumbar spine, which corresponds to an effect size of |δ| = 1.5.^5^ We therefore needed a sample size of 11 in each group to reliably (with probability ≥0.9) detect an effect size of |δ| ≥ 1.5, assuming a two‐sided criterion for detection that allows for a maximum Type I error rate of α = 0.05. We finally aimed to conservatively include 25 patients in both groups to account for missing data.

### Statistical analysis

Group comparisons were performed with analysis of variance post hoc tests with *P* value adjusted with Holm method. Categorical variables were compared with the chi‐squared test. All differences were considered significant when *P* value < 0.05. Between‐group percentages and absolute changes in BMD and BTMs were assessed with a mixed model for repeated measures (MMRM) using Satterthwaite and restricted maximum likelihood method with treatment sequence, time, treatment‐by‐time interaction, and baseline BMD as fixed effects and with patients as random effect. Sensitivity analyses missing data (if <5%) were imputed using a random forest algorithm (Orange, version 3.37.0). The imputation process was iterative and used a random forest model with 10 trees; the model allowed for an unlimited number of features and unrestricted tree depth. Splitting of nodes continued until each node contained fewer than five instances. To assess the robustness of the primary propensity score–matched analysis, we conducted a sensitivity analysis using inverse probability of treatment weighting (IPTW) based on the full cohort (n = 388 Dmab only, n = 25 Dmab + Romo). Propensity scores were estimated through logistic regression, including the same covariates used for matching (age, sex, T score at femoral neck, total hip and lumbar spine, prior vertebral and nonvertebral fractures, P1nP and CTX serum levels). Stabilized inverse probability weights were applied to everyone to create a pseudopopulation in which baseline covariates were balanced between the two groups. To reduce the impact of extreme weights, we performed trimming of propensity scores at the first and 99th percentiles. Covariate balance after weighting was assessed by standardized mean differences, with values <0.10 considered indicative of good balance. Weighted analyses were then conducted using MMRM for repeated measures to estimate the treatment effect on BMD and BTMs similarly to the primary analysis. All statistical analyses were performed using SPSS Version 26 (SPSS, Inc.), GraphPad Prism version 9.5.1 (GraphPad Software), JASP (version 0.19.0), and R (version 4.5.1). This study was approved by the University of Verona ethic committee (prot. registration: REUMABANK 1483CESC). All patients provided informed consent to participate in the study and/or retrospective collection of the data. Patients provided consent for publication of anonymized data. Data of the analysis is available upon reasonable request.

## RESULTS

Fifty patients were included in the prospective study, and data before inclusion were retrieved from electronic medical records. Supplementary Figure S1 shows the study flowchart. Twenty‐five women received Dmab alone, and 25 women received Dmab first and then had Romo added to ongoing Dmab (Dmab + Romo). Characteristics of the population included are shown in Table 1.

**Table 1:** Study population characteristics*

Characteristics	Denosumab to denosumab + romosozumab (n = 25)	Matched denosumab to denosumab (n = 25)	Unmatched denosumab to denosumab (n = 388)	Abs. SMD Pre‐matching or Abs. Diff. Pre, %	Abs. SMD Post‐matching or Abs. Diff. Post, %
Age, mean ± SD, y	73.5 ± 8.9	74.1 ± 9.1	68.2 ± 10.5	0.51	0.07
BMI, mean ± SD	24.6 ± 2.9	24.4 ± 3.0	24.8 ± 3.2	0.06	0.07
Comorbidities, n (%)					
Rheumatoid arthritis	1 (4)	1 (4)	28 (7)	3	0
MACE	0 (0)	1 (4)	28 (7)	7	4
Hypertension	14 (56)	15 (60)	252 (65)	9	4
VTE	0 (0)	3 (12)	31 (8)	8	12
CKD	2 (8)	3 (12)	58 (15)	7	4
MGUS	0 (0)	1 (4)	12 (3)	3	4
Prevalent vertebral fractures, n (%)	24 (96)	23 (92)	310 (80)	16	4
Recent vertebral fractures (<2 y), n (%)	13 (52)	12 (48)	116 (30)	22	4
Nonvertebral MOF fractures, n (%)	16 (64)	15 (60)	155 (40)	24	4
Hip fractures, n (%)	1 (4)	1 (4)	12 (3)	1	0
M−24 BMD and markers					
Lumbar spine BMD, mean ± SD, g/cm^2^	0.811 ± 0.113	0.801 ± 0.120	0.850 ± 0.130	0.3	0.09
Femoral neck BMD, mean ± SD, g/cm^2^	0.659 ± 0.169	0.670 ± 0.167	0.700 ± 0.180	0.23	0.07
Total hip BMD, mean ± SD, g/cm^2^	0.686 ± 0.187	0.690 ± 0.179	0.720 ± 0.190	0.18	0.02
Lumbar spine T score, mean ± SD	−1.9 ± 0.9	−1.9 ± 1.0	−1.6 ± 1.1	0.28	0.01
Femoral neck T score, mean ± SD	−1.9 ± 1.0	−2.0 ± 1.0	−1.7 ± 1.2	0.17	0.01
Total hip T score, mean ± SD	−2.3 ± 1.2	−2.3 ± 1.1	−2.0 ± 1.3	0.23	0.01
CTX, mean ± SD, ng/mL	0.387 ± 0.195	0.375 ± 0.200	0.410 ± 0.220	0.11	0.06
P1nP, mean ± SD, ng/mL	81.2 ± 22.7	82.5 ± 23.5	75.0 ± 25.0	0.25	0.07
PTH, mean ± SD, pg/mL	44.1 ± 18.1	42.5 ± 18.8	40.0 ± 20.0	0.21	0.09
Vitamin D, mean ± SD, ng/mL	38.1 ± 13.5	39.0 ± 13.8	35.0 ± 15.0	0.21	0.07
M0 BMD and markers					
Lumbar spine BMD, mean ± SD, g/cm^2^	0.853 ± 0.123	0.845 ± 0.131	0.891 ± 0.170	0.23	0.06
Femoral neck BMD, mean ± SD, g/cm^2^	0.673 ± 0.175	0.685 ± 0.174	0.712 ± 0.190	0.21	0.07
Total hip BMD, mean ± SD, g/cm^2^	0.702 ± 0.194	0.696 ± 0.187	0.731 ± 0.201	0.15	0.03
Lumbar spine T score, mean ± SD	−1.4 ± 0.7	−1.4 ± 0.9	−1.1 ± 1.0	0.30	<0.01
Femoral neck T score, mean ± SD	−1.8 ± 1.0	−1.9 ± 1.1	−1.3 ± 0.9	0.55	0.09
Total hip T score, mean ± SD	−2.2 ± 1.1	−2.2 ± 1.1	−1.7 ± 1.2	0.41	<0.01
CTX, mean ± SD, ng/mL	0.120 ± 0.081	0.113 ± 0.078	0.093 ± 0.070	0.38	0.08
P1nP, mean ± SD, ng/mL	32.1 ± 21.4	34.2 ± 22.3	36.6 ± 20.3	0.22	0.09
PTH, mean ± SD, pg/mL	49.1 ± 21.1	48.5 ± 22.8	45.0 ± 20.0	0.20	0.03
Vitamin D, mean ± SD, ng/mL	41.1 ± 16.5	42.0 ± 17.8	35.0 ± 15.0	0.40	0.05

* Abs. diff., absolute difference; BMD, bone mineral density; BMI, body mass index; CKD, chronic kidney disease stages 3a–3b by KDIGO guidelines; CTX, C‐terminal telopeptide of type I collagen; Dmab, denosumab; M, month; MACE, major adverse cardiovascular events; MGUS, monoclonal gammopathy of undetermined significance; MOF, major osteoporotic fractures; P1nP, procollagen I intact N‐terminal peptide; PTH, parathyroid hormone; Romo, romosozumab; SMD, standardized mean difference; VTE, venous thromboembolism.

### Retrospective study results (M−24 to M0)

**Figure 2:**
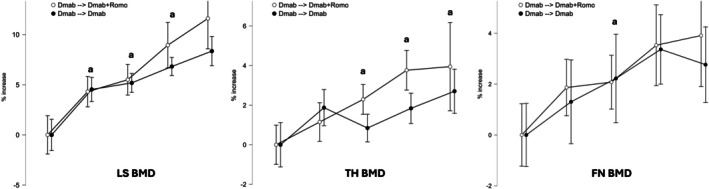
BMD changes over the study period for LS (left), TH (middle), and FN (right). Between‐group differences were tested with a mixed model for repeated measures using Satterthwaite and restricted maximum likelihood method with treatment sequence, time, treatment‐by‐time interaction, and baseline BMD as fixed effects and with patients as random effect. a, *P* < 0.05 versus baseline; b, *P* = 0.074 for Dmab ➔ Dmab versus Dmab ➔ Dmab + Romo. Error bars show 95% confidence interval. BMD, bone mineral density; Dmab, denosumab; FN, femoral neck; LS, lumbar spine; Romo, romosozumab; TH, total hip.

**Figure 3:**
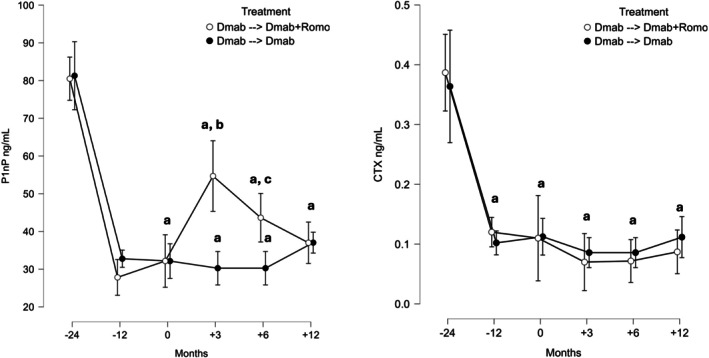
Bone turnover markers changes over the study period. P1np (left) and CTX (right). Between‐group differences were tested with a mixed model for repeated measures using Satterthwaite and restricted maximum likelihood method with treatment sequence, time, treatment‐by‐time interaction, and baseline bone mineral density as fixed effects and with patients as random effect. a, *P* < 0.05 versus baseline; b, *P* < 0.001 for Dmab ➔ Dmab versus Dmab ➔ Dmab + Romo; c, *P* = 0.057 for Dmab ➔ Dmab versus Dmab ➔ Dmab + Romo. Error bars show 95% confidence interval. CTX, C‐terminal telopeptide of type I collagen; Dmab, Dmab; P1nP, procollagen I intact N‐terminal peptide; Romo, Romo.

BMD and BTMs changes from M−24 and M0 are shown in Figures 2 and 3, respectively. BMD significantly increased in both groups between M−24 and M0. Lumbar spine increased by 5.2% (95% confidence interval [CI] 3.8–6.6; *P* < 0.001) and 5.5% (95% CI 3.9–7.1; *P* < 0.001), femoral neck increased by 2.1% (95% CI 0.9–3.3; *P* = 0.028) and 2.2% (95% CI 1.0–3.4; *P* = 0.027), and total hip increased by 2.3% (95% CI 1.3–3.3; *P* < 0.001) and 0.8% (95% CI −0.2 to 1.8; *P* = nonsignificant [ns]).

We found a statistically significant decrease in P1nP levels from M−24 to M−12 (−48.5 ng/mL, 95% CI −54.4 to −42.6; *P* < .001 and −52.7 ng/mL, 95% CI −58.2 to −47.2; *P* < .001) and from M−24 to M0 (−49.1 ng/mL, 95% CI −61.8 to −36.4; *P* < 0.001 and −48.3 ng/mL, 95% CI −60.3 to −36.3; *P* < 0.001). CTX decreased significantly in all Dmab users at M−12 (−0.267 ng/mL, 95% CI −0.31 to −0.22; *P* < 0.001 and −0.262 ng/mL, 95% CI −0.32 to −0.21; *P* < 0.001) and remained suppressed thereafter.

### Prospective study results (M0 to M+12)

BMD and BTMs changes from M−24 and M0 are shown in Figures 2 and 3, respectively.

### Dmab‐alone continued

BMD slightly increased at all sites between M0 and M+12 but did not reach statistical significance at any site. Between M−24 and M+12, the overall increases in the Dmab‐alone group were as follows: lumbar spine, 8.3% (95% CI 1.8–14.8; *P* = 0.042); femoral neck, 2.8% (95% CI −1.9 to 7.5; *P* = ns); and total hip, 2.7% (95% CI −1.4 to 6.8; *P* = ns). P1nP and CTX remained stable between M0 and M+12.

### Dmab + Romo

BMD significantly increased at lumbar spine between M0 and M+6 (3.4%; 95% CI 0.1–6.7; *P* = 0.034) and between M0 and M+12 (6.1%; 95% CI 0.6–11.6; *P* = 0.028). Between M−24 and M+12, the overall increases in the Dmab + Romo group were lumbar spine 11.6% (95% CI 5.7–17.5; *P* = 0.010), femoral neck 3.9% (95% CI 0.1–9.6; *P* = 0.042), and total hip 3.9% (95% CI −0.1 to 7.6; *P* = 0.054).

P1nP increased significantly between M0 and M+3 (+22.5 ng/mL, 95% CI 5.4–39.6; *P* = 0.028). P1nP decreased thereafter (−17.6 ng/mL, 95% CI −37.4 to 2.2; *P* = ns at M+12). CTX did not change between M0 and M+12.

### Group comparisons

In the MMRM, we found a trend toward significance at lumbar spine M+12 between the Dmab + Romo and Dmab‐alone groups (difference between groups 3.2%, 95% CI −5.4 to 11.8; *P* = 0.074). We did not find any significant difference in femoral neck and total hip between groups at M+12.

We found a significant difference at M+3 in P1nP levels between the Dmab‐alone and Dmab + Romo groups (difference between groups 24.4 ng/mL, 95% CI 0.3–49.1; *P* = 0.033). We found a trend toward significance at M+6 in P1nP levels between the Dmab‐alone and Dmab + Romo groups (difference between groups 13.4 ng/mL, 95% CI −1.7 to 28.5; *P* = 0.057). In the sensitivity analysis using IPTW, after weighing, baseline characteristics were well balanced (all standardized mean differences <0.1), similar to the propensity score matching.

The weighted analysis confirmed a trend toward greater lumbar spine BMD gain at M+12 in the Dmab + Romo group compared to Dmab alone: mean difference 3.1% (95% CI −1.5 to 7.5; *P* = 0.064). Similar trends were observed at the femoral neck (difference 1.0%, 95% CI −1.8 to 3.9) and total hip (difference 1.2%, 95% CI −1.3 to 3.8). For BTMs, P1nP at M+3 remained significantly higher in the combination group (difference 21.0 ng/mL, 95% CI 5.0–37.0; *P* = 0.022), consistent with the primary analysis.

## DISCUSSION

Herein we investigated the effects of Romo in combination with ongoing Dmab treatment in postmenopausal women with severe osteoporosis. In aggregate, we showed that ongoing Dmab did not blunt the anabolic response to Romo.

When evaluating the percentage change from M−24 (24 months before initiation of Romo) to M+12, the final BMD gains were around 11%. In contrast, the Dmab‐only group exhibited a modest, nonsignificant, 3% increase in BMD between M0 and M+12, leading to a total gain of 8% over the 36‐month study period. However, the between‐group difference at the lumbar spine was modest (3.3%, 95% CI −5.2 to 11.8), and due to the limited sample size, the study was underpowered to detect the expected 6% difference used for sample size estimation. At the femoral neck and total hip, the patterns were similar, albeit of a smaller magnitude, with the combination group showing greater BMD response than the Dmab‐only group.

The analysis of BTMs adds another layer of understanding to the anabolic effects of Romo. After the initiation of Romo in the combination group, P1nP increased markedly, with a similar magnitude to what has been reported in patients on Romo naive to treatment,^5^, ^10^ indicating a preserved anabolic response despite continued CTX suppression. Interestingly, we previously demonstrated that Dmab and bisphosphonates increased circulating sclerostin levels, supporting the opportunity of a combined approach with Romo to boost the BMD response.^6^, ^11^ In aggregate, our finding suggests that Romo can stimulate bone formation through modeling‐based mechanisms even in the context of continued Dmab treatment. Indeed, the deep suppression of CTX in this group further indicates a net anabolic effect with new bone mass created. Interestingly, a recently published study showed that, in mice, Dmab did not blunt the anabolic modeling‐based effect of Romo, with overall similar BMD findings compared to our study.^12^ The greater BMD gains reported in naive patients receiving Romo^10^, ^13^ can be attributed to the simultaneous stimulation of modeling‐based and remodeling‐based bone formation. The latter was indeed not fully engaged in patients who had already experienced remodeling suppression during Dmab therapy. Indeed, the resorptions cavities were already filled during the first period on Dmab (M−24 to M0), and, therefore, the BMD gain, in terms of remodeling‐based bone formation, were already accrued in those patients.

In our study we included patients who had been on Dmab for a long time to ensure they were in the fully closed remodeling phase, where the response to ongoing Dmab is consistent, and any additional BMD changes did not reflect remodeling‐based bone formation but only modeling‐based bone formation as observed in the extension phase of the FREEDOM trial.^14^ Moreover, the choice of a two‐year prior Dmab exposure was mainly pragmatic, aiming to ensure a homogeneous cohort, and we believe that a longer prior exposure would not have significantly altered the outcomes. Indeed, although the treatment duration's influence is critical in the context of discontinuation (rebound risk), it should not affect the therapeutic response to combination therapy with Romo in this specific study.

Combining Dmab with anabolic treatments has already been done in the literature. Our group previously showed that Romo combined with ongoing Dmab for 6 months increased BMD and P1nP more than Dmab alone^5^; herein we presented the 12‐month findings with additional insight on the BMD and BTMs trends before Romo treatment initiation. The efficacy of combining teriparatide and Dmab was reported in the Dmab and Teriparatide Administration (DATA) study.^15^, ^16^ However, the results of the DATA study and ours should not be directly compared. Indeed, the DATA study included only Dmab‐naive patients. Teriparatide acts primarily through stimulating remodeling‐based bone formation and needs opened remodeling units to be maximally effective on BMD. It could be speculated, therefore, that in patients with fully closed remodeling surfaces due to long‐term Dmab therapy, the addition of teriparatide would lose part or even most of its efficacy. Another study exploring combination of treatments was conducted by our group in 2016.^17^ In that study, teriparatide was introduced three months after Dmab initiation with superimposable results to the DATA study. Nevertheless, the short three‐month period on Dmab before teriparatide addition may be not sufficient to fully close all remodeling units, and the reversal phase may still have been incomplete. In contrast, in the present study Dmab was administered for two years, and, therefore, we can speculate that the P1nP and BMD increases were mainly due to modeling‐based bone formation, which was not affected by ongoing Dmab treatment. Kumar and colleagues recently published a small case series of three patients in whom Romo was added to Dmab after three months from the last Dmab dose.^18^ Romo was then continued for 12 months, and Dmab was restarted after 9 months from the last dose. In this scheme the overlap between the two drugs was shorter, with three months off Dmab between month 3 and month 6 of Romo treatment. Despite the small number of patients included, the authors found somehow similar results to ours, with larger increases at the lumbar spine. The off‐Dmab period might permit a reopening of the bone remodeling units with greater efficacy of Romo (ie, overflow remodeling‐based bone formation). However, this strategy may not be optimal. First, a slight decrease in BMD for the reopening of remodeling units with subsequent refill would not really increase bone mass but just merely play with the remodeling space. Second, prior studies showed that Romo cannot effectively prevent the rebound associated Dmab withdrawal.^3^, ^4^, ^19^, ^20^, ^21^ Indeed, in the study by Kumar et al, CTX serum levels steeply increased above the normal postmenopausal range despite Romo treatment.^18^ More recently, Hong and colleagues showed that the transition from Dmab to Romo resulted in a significant increase in lumbar spine BMD but not total hip BMD.^22^ Of note, patients were treated for a median period of two years with Dmab, limiting the generalizability to short‐term Dmab users. More importantly, Hong and colleagues reported a significant increase in CTX serum levels (greater than +100% at month 12) that paralleled the somewhat similar P1nP increase, after the transition from Dmab to Romo. Nonetheless, even if the combination might represent an effective strategy, our results should consider broader clinical and economic contexts, as the combination is significantly more costly and might not be fully reimbursed by many health systems.

The more pronounced BMD gains observed at the lumbar spine compared to the femoral neck and total hip likely reflect site‐specific differences in bone composition and responsiveness to Romo. The lumbar spine, with its higher trabecular content, is particularly sensitive to modeling‐based bone formation, whereas cortical sites such as the femoral neck and hip rely more on remodeling‐based bone formation and overflow remodeling‐based bone formation with closure of cortical porosity. In our cohort, the prolonged suppression of bone turnover induced by prior Dmab therapy may have attenuated the remodeling response at these cortical‐rich sites. Consistently, Kashii et al reported that hip BMD increases with Romo were observed only in patients with normal or elevated baseline P1nP levels, highlighting the role of active bone formation in driving gains at the femoral neck and hip.^23^

Our study should be interpreted in light of the strengths and limitations. The major strength is the detailed collection of data in the prospective phase of the study with single‐batch analysis of BTMs and systematic collection of BMD data. Albeit potentially of scientific interest, we did not include a group that switched from Dmab to Romo. Indeed, we felt that this strategy would not have been ethical due to the potential risk of rebound effect upon Dmab discontinuation. It has been shown that Romo does not totally prevent the rebound in BTMs associated with Dmab discontinuation, and there are reports of multiple vertebral fractures in patients transitioning from Dmab to Romo.^3^, ^4^ However, Hong et al reported no vertebral fractures in 86 patients who transitioned to Romo after a median of four Dmab doses.^22^ Yet, this finding should be interpreted with caution. The absence of fractures in that study could be influenced by factors such as the shorter duration of prior Dmab treatment and chance, rather than providing definitive evidence against the risk of rebound fractures. We also acknowledge that body mass index (BMI) and renal function (eGFR) were not included in the propensity score model or IPTW, although patients with severe chronic kidney disease were excluded and BMI was well balanced between groups; residual confounding from unmeasured variables cannot be completely ruled out. Further adequately powered randomized controlled trials are needed to confirm our findings; however, a randomized controlled trial design was not feasible in our setting due to ethical considerations. Our study may therefore serve as a basis for future sample size estimations. The lack of fracture outcomes is another limitation of our study, and future studies with larger cohorts are needed to evaluate this endpoint.

In conclusion, our data suggest that Romo can retain its anabolic potential when introduced in combination with Dmab. The findings support the combination of Romo and Dmab in patients with severe osteoporosis treated with long‐term Dmab in whom the response to treatment is not deemed adequate. Yet, these findings should be interpreted as exploratory and hypothesis‐generating, pending confirmation in larger, prospective studies.

## AUTHOR CONTRIBUTIONS

All authors contributed to at least one of the following manuscript preparation roles: conceptualization AND/OR methodology, software, investigation, formal analysis, data curation, visualization, and validation AND drafting or reviewing/editing the final draft. As corresponding author, Dr Adami confirms that all authors have provided the final approval of the version to be published and takes responsibility for the affirmations regarding article submission (eg, not under consideration by another journal), the integrity of the data presented, and the statements regarding compliance with institutional review board/Declaration of Helsinki requirements.

## Supporting information


**Disclosure form**.

**Appendix S1:** Supplementary Information.
