# Supplementary material for: Romosozumab and Denosumab Combination Therapy After Denosumab in Postmenopausal Osteoporosis
Source: Arthritis Rheumatol. 2026 Feb 22;78(5):1176–83. doi: 10.1002/art.70002 (PMC13129633; doi:10.1002/art.70002)
Supplement: Supplementary file 1 — Disclosure form. [file ART-78-1176-s001.pdf]

ICMJE DISCLOSURE FORM

Date:____30 Oct 2025____
Your Name: Giovanni Adami
Manuscript Title: Romosozumab and Denosumab Combination Therapy After Denosumab in Postmenopausal Osteoporosis
Manuscript number (if known):____

In the interest of transparency, we ask you to disclose all relationships/activities/interests listed below that are related to the content of your manuscript. "Related" means any relation with for-profit or not-for-profit third parties whose interests may be affected by the content of the manuscript. Disclosure represents a commitment to transparency and does not necessarily indicate a bias. If you are in doubt about whether to list a relationship/activity/interest, it is preferable that you do so.

The following questions apply to the author's relationships/activities/interests as they relate to the current manuscript only.

The author's relationships/activities/interests should be defined broadly. For example, if your manuscript pertains to the epidemiology of hypertension, you should declare all relationships with manufacturers of antihypertensive medication, even if that medication is not mentioned in the manuscript.

In item #1 below, report all support for the work reported in this manuscript without time limit. For all other items, the time frame for disclosure is the past 36 months.

		**Name all entities with whom you have this relationship or indicate none (add rows as needed)**	**Specifications/Comments (e.g., if payments were made to you or to your institution)**
		**Time frame: Since the initial planning of the work**	
1	All support for the present manuscript (e.g., funding, provision of study materials, medical writing, article processing charges, etc.) **No time limit for this item.**	X None	
		**Time frame: past 36 months**	
2	Grants or contracts from any entity (if not indicated in item #1 above).	X None	
3	Royalties or licenses	X None	

4	Consulting fees	_X___ None	
5	Payment or honoraria for lectures, presentations, speakers bureaus, manuscript writing or educational events	____ None	Theramex, UCB, Lilly, Galapagos, Fresenius Kabi, Amgen, BMS,
			Abiogen and Pfizer
6	Payment for expert testimony	X None	
7	Support for attending meetings and/or travel	X None	
8	Patents planned, issued or pending	X None	
9	Participation on a Data Safety Monitoring Board or Advisory Board	X None	
10	Leadership or fiduciary role in other board, society, committee or advocacy group, paid or unpaid	X None	
11	Stock or stock options	X None	
12	Receipt of equipment, materials, drugs, medical writing, gifts or other services	X None	
13	Other financial or non-financial interests	X None	

Please place an "X" next to the following statement to indicate your agreement:

_X_ I certify that I have answered every question and have not altered the wording of any of the questions on this form.

ICMJE DISCLOSURE FORM

Date:____30 Oct 2025____
Your Name:____Carmen Dartizio____
Manuscript Title:_ Romosozumab and Denosumab Combination Therapy After Denosumab in Postmenopausal Osteoporosis
Manuscript number (if known):____

In the interest of transparency, we ask you to disclose all relationships/activities/interests listed below that are related to the content of your manuscript. "Related" means any relation with for-profit or not-for-profit third parties whose interests may be affected by the content of the manuscript. Disclosure represents a commitment to transparency and does not necessarily indicate a bias. If you are in doubt about whether to list a relationship/activity/interest, it is preferable that you do so.

The following questions apply to the author's relationships/activities/interests as they relate to the current manuscript only.

The author's relationships/activities/interests should be defined broadly. For example, if your manuscript pertains to the epidemiology of hypertension, you should declare all relationships with manufacturers of antihypertensive medication, even if that medication is not mentioned in the manuscript.

In item #1 below, report all support for the work reported in this manuscript without time limit. For all other items, the time frame for disclosure is the past 36 months.

		**Name all entities with whom you have this relationship or indicate none (add rows as needed)**	**Specifications/Comments (e.g., if payments were made to you or to your institution)**
		**Time frame: Since the initial planning of the work**	
1	All support for the present manuscript (e.g., funding, provision of study materials, medical writing, article processing charges, etc.) **No time limit for this item.**	_X___ None	
		**Time frame: past 36 months**	
2	Grants or contracts from any entity (if not indicated in item #1 above).	__ X __ None	
3	Royalties or licenses	_ X ___ None	

4	Consulting fees	_ X ___ None	
5	Payment or honoraria for lectures, presentations, speakers bureaus, manuscript writing or educational events	_ X ___ None	
6	Payment for expert testimony	_ X ___ None	
7	Support for attending meetings and/or travel	_ X ___ None	
8	Patents planned, issued or pending	_ X ___ None	
9	Participation on a Data Safety Monitoring Board or Advisory Board	_ X ___ None	
10	Leadership or fiduciary role in other board, society, committee or advocacy group, paid or unpaid	_ X ___ None	
11	Stock or stock options	___ X _ None	
12	Receipt of equipment, materials, drugs, medical writing, gifts or other services	__ X _ None	
13	Other financial or non-financial interests	_ X ___ None	

Please place an "X" next to the following statement to indicate your agreement:

_ X __ I certify that I have answered every question and have not altered the wording of any of the questions on this form.

ICMJE DISCLOSURE FORM

Date:____30 Oct 2025____
Your Name:____Emma Pasetto____
Manuscript Title:_ Romosozumab and Denosumab Combination Therapy After Denosumab in Postmenopausal Osteoporosis
Manuscript number (if known):____

In the interest of transparency, we ask you to disclose all relationships/activities/interests listed below that are related to the content of your manuscript. "Related" means any relation with for-profit or not-for-profit third parties whose interests may be affected by the content of the manuscript. Disclosure represents a commitment to transparency and does not necessarily indicate a bias. If you are in doubt about whether to list a relationship/activity/interest, it is preferable that you do so.

The following questions apply to the author's relationships/activities/interests as they relate to the <u>current manuscript only</u>.

The author's relationships/activities/interests should be <u>defined broadly</u>. For example, if your manuscript pertains to the epidemiology of hypertension, you should declare all relationships with manufacturers of antihypertensive medication, even if that medication is not mentioned in the manuscript.

In item #1 below, report all support for the work reported in this manuscript without time limit. For all other items, the time frame for disclosure is the past 36 months.

		Name all entities with whom you have this relationship or indicate none (add rows as needed)	Specifications/Comments (e.g., if payments were made to you or to your institution)
		**Time frame: Since the initial planning of the work**	
1	All support for the present manuscript (e.g., funding, provision of study materials, medical writing, article processing charges, etc.) **No time limit for this item.**	_X___ None	
		**Time frame: past 36 months**	
2	Grants or contracts from any entity (if not indicated in item #1 above).	__ X __ None	
3	Royalties or licenses	_ X ___ None	

4	Consulting fees	_ **X** ___ None	
5	Payment or honoraria for lectures, presentations, speakers bureaus, manuscript writing or educational events	_ **X** ___ None	
6	Payment for expert testimony	_ **X** ___ None	
7	Support for attending meetings and/or travel	_ **X** ___ None	
8	Patents planned, issued or pending	_ **X** ___ None	
9	Participation on a Data Safety Monitoring Board or Advisory Board	_ **X** ___ None	
10	Leadership or fiduciary role in other board, society, committee or advocacy group, paid or unpaid	_ **X** ___ None	
11	Stock or stock options	___ **X** _ None	
12	Receipt of equipment, materials, drugs, medical writing, gifts or other services	__ **X** __ None	
13	Other financial or non-financial interests	_ **X** ___ None	

Please place an "X" next to the following statement to indicate your agreement:

_ X __ I certify that I have answered every question and have not altered the wording of any of the questions on this form.

ICMJE DISCLOSURE FORM

Date:____30 Oct 2025____
Your Name:____Camilla Benini____
Manuscript Title:_ Romosozumab and Denosumab Combination Therapy After Denosumab in Postmenopausal Osteoporosis
Manuscript number (if known):____

In the interest of transparency, we ask you to disclose all relationships/activities/interests listed below that are related to the content of your manuscript. "Related" means any relation with for-profit or not-for-profit third parties whose interests may be affected by the content of the manuscript. Disclosure represents a commitment to transparency and does not necessarily indicate a bias. If you are in doubt about whether to list a relationship/activity/interest, it is preferable that you do so.

The following questions apply to the author's relationships/activities/interests as they relate to the current manuscript only.

The author's relationships/activities/interests should be defined broadly. For example, if your manuscript pertains to the epidemiology of hypertension, you should declare all relationships with manufacturers of antihypertensive medication, even if that medication is not mentioned in the manuscript.

In item #1 below, report all support for the work reported in this manuscript without time limit. For all other items, the time frame for disclosure is the past 36 months.

		**Name all entities with whom you have this relationship or indicate none (add rows as needed)**	**Specifications/Comments (e.g., if payments were made to you or to your institution)**
**Time frame: Since the initial planning of the work**			
1	All support for the present manuscript (e.g., funding, provision of study materials, medical writing, article processing charges, etc.) **No time limit for this item.**	_X___ None	
**Time frame: past 36 months**			
2	Grants or contracts from any entity (if not indicated in item #1 above).	__ X __ None	
3	Royalties or licenses	_ X ___ None	

4	Consulting fees	_ X ___ None	
5	Payment or honoraria for lectures, presentations, speakers bureaus, manuscript writing or educational events	_ X ___ None	
6	Payment for expert testimony	_ X ___ None	
7	Support for attending meetings and/or travel	_ X ___ None	
8	Patents planned, issued or pending	_ X ___ None	
9	Participation on a Data Safety Monitoring Board or Advisory Board	_ X ___ None	
10	Leadership or fiduciary role in other board, society, committee or advocacy group, paid or unpaid	_ X ___ None	
11	Stock or stock options	___ X _ None	
12	Receipt of equipment, materials, drugs, medical writing, gifts or other services	__ X __ None	
13	Other financial or non-financial interests	_ X ___ None	

Please place an “X” next to the following statement to indicate your agreement:

_ X __ I certify that I have answered every question and have not altered the wording of any of the questions on this form.

ICMJE DISCLOSURE FORM

Date:____30 Oct 2025____
Your Name:____Anna Piccinelli____
Manuscript Title:_ Romosozumab and Denosumab Combination Therapy After Denosumab in Postmenopausal Osteoporosis
Manuscript number (if known):____

In the interest of transparency, we ask you to disclose all relationships/activities/interests listed below that are related to the content of your manuscript. "Related" means any relation with for-profit or not-for-profit third parties whose interests may be affected by the content of the manuscript. Disclosure represents a commitment to transparency and does not necessarily indicate a bias. If you are in doubt about whether to list a relationship/activity/interest, it is preferable that you do so.

The following questions apply to the author's relationships/activities/interests as they relate to the current manuscript only.

The author's relationships/activities/interests should be defined broadly. For example, if your manuscript pertains to the epidemiology of hypertension, you should declare all relationships with manufacturers of antihypertensive medication, even if that medication is not mentioned in the manuscript.

In item #1 below, report all support for the work reported in this manuscript without time limit. For all other items, the time frame for disclosure is the past 36 months.

		**Name all entities with whom you have this relationship or indicate none (add rows as needed)**	**Specifications/Comments (e.g., if payments were made to you or to your institution)**
		**Time frame: Since the initial planning of the work**	
1	All support for the present manuscript (e.g., funding, provision of study materials, medical writing, article processing charges, etc.) **No time limit for this item.**	_X___ None	
		**Time frame: past 36 months**	
2	Grants or contracts from any entity (if not indicated in item #1 above).	__ X __ None	
3	Royalties or licenses	_ X ___ None	

4	Consulting fees	_ X ___ None	
5	Payment or honoraria for lectures, presentations, speakers bureaus, manuscript writing or educational events	_ X ___ None	
6	Payment for expert testimony	_ X ___ None	
7	Support for attending meetings and/or travel	_ X ___ None	
8	Patents planned, issued or pending	_ X ___ None	
9	Participation on a Data Safety Monitoring Board or Advisory Board	_ X ___ None	
10	Leadership or fiduciary role in other board, society, committee or advocacy group, paid or unpaid	_ X ___ None	
11	Stock or stock options	___ X _ None	
12	Receipt of equipment, materials, drugs, medical writing, gifts or other services	__ X __ None	
13	Other financial or non-financial interests	_ X ___ None	

Please place an "X" next to the following statement to indicate your agreement:

_ X __ I certify that I have answered every question and have not altered the wording of any of the questions on this form.

ICMJE DISCLOSURE FORM

Date:____30 Oct 2025____
Your Name: Filippo Montanari
Manuscript Title: Romosozumab and Denosumab Combination Therapy After Denosumab in Postmenopausal Osteoporosis
Manuscript number (if known):____

In the interest of transparency, we ask you to disclose all relationships/activities/interests listed below that are related to the content of your manuscript. "Related" means any relation with for-profit or not-for-profit third parties whose interests may be affected by the content of the manuscript. Disclosure represents a commitment to transparency and does not necessarily indicate a bias. If you are in doubt about whether to list a relationship/activity/interest, it is preferable that you do so.

The following questions apply to the author's relationships/activities/interests as they relate to the current manuscript only.

The author's relationships/activities/interests should be defined broadly. For example, if your manuscript pertains to the epidemiology of hypertension, you should declare all relationships with manufacturers of antihypertensive medication, even if that medication is not mentioned in the manuscript.

In item #1 below, report all support for the work reported in this manuscript without time limit. For all other items, the time frame for disclosure is the past 36 months.

		**Name all entities with whom you have this relationship or indicate none (add rows as needed)**	**Specifications/Comments (e.g., if payments were made to you or to your institution)**
		**Time frame: Since the initial planning of the work**	
1	All support for the present manuscript (e.g., funding, provision of study materials, medical writing, article processing charges, etc.) **No time limit for this item.**	X None	
		**Time frame: past 36 months**	
2	Grants or contracts from any entity (if not indicated in item #1 above).	X None	
3	Royalties or licenses	X None	

4	Consulting fees	_X_ None	
5	Payment or honoraria for lectures, presentations, speakers bureaus, manuscript writing or educational events	_X_ None	
6	Payment for expert testimony	_X_ None	
7	Support for attending meetings and/or travel	_X_ None	
8	Patents planned, issued or pending	_X_ None	
9	Participation on a Data Safety Monitoring Board or Advisory Board	_X_ None	
10	Leadership or fiduciary role in other board, society, committee or advocacy group, paid or unpaid	_X_ None	
11	Stock or stock options	_X_ None	
12	Receipt of equipment, materials, drugs, medical writing, gifts or other services	_X_ None	
13	Other financial or non-financial interests	_X_ None	

Please place an "X" next to the following statement to indicate your agreement:

_X_ I certify that I have answered every question and have not altered the wording of any of the questions on this form.

ICMJE DISCLOSURE FORM

Date:____30 Oct 2025____
Your Name:_ Angelo Fassio**____**
Manuscript Title: Romosozumab and Denosumab Combination Therapy After Denosumab in Postmenopausal Osteoporosis
Manuscript number (if known):____

In the interest of transparency, we ask you to disclose all relationships/activities/interests listed below that are related to the content of your manuscript. "Related" means any relation with for-profit or not-for-profit third parties whose interests may be affected by the content of the manuscript. Disclosure represents a commitment to transparency and does not necessarily indicate a bias. If you are in doubt about whether to list a relationship/activity/interest, it is preferable that you do so.

The following questions apply to the author's relationships/activities/interests as they relate to the current manuscript only.

The author's relationships/activities/interests should be defined broadly. For example, if your manuscript pertains to the epidemiology of hypertension, you should declare all relationships with manufacturers of antihypertensive medication, even if that medication is not mentioned in the manuscript.

In item #1 below, report all support for the work reported in this manuscript without time limit. For all other items, the time frame for disclosure is the past 36 months.

		**Name all entities with whom you have this relationship or indicate none (add rows as needed)**	**Specifications/Comments (e.g., if payments were made to you or to your institution)**
**Time frame: Since the initial planning of the work**			
1	All support for the present manuscript (e.g., funding, provision of study materials, medical writing, article processing charges, etc.) **No time limit for this item.**	X None	
**Time frame: past 36 months**			
2	Grants or contracts from any entity (if not indicated in item #1 above).	X None	
3	Royalties or licenses	X None	

4	Consulting fees	X None	
5	Payment or honoraria for lectures, presentations, speakers bureaus, manuscript writing or educational events	____ None	Abiogen, Novartis, Neopharmed
6	Payment for expert testimony	X None	
7	Support for attending meetings and/or travel	X None	
8	Patents planned, issued or pending	X None	
9	Participation on a Data Safety Monitoring Board or Advisory Board	X None	
10	Leadership or fiduciary role in other board, society, committee or advocacy group, paid or unpaid	X None	
11	Stock or stock options	X None	
12	Receipt of equipment, materials, drugs, medical writing, gifts or other services	X None	
13	Other financial or non-financial interests	X None	

Please place an "X" next to the following statement to indicate your agreement:

_X_ I certify that I have answered every question and have not altered the wording of any of the questions on this form.

ICMJE DISCLOSURE FORM

Date:____30 Oct 2025____
Your Name:___ Ombretta Viapiana**____**
Manuscript Title: Romosozumab and Denosumab Combination Therapy After Denosumab in Postmenopausal Osteoporosis
Manuscript number (if known):____

In the interest of transparency, we ask you to disclose all relationships/activities/interests listed below that are related to the content of your manuscript. "Related" means any relation with for-profit or not-for-profit third parties whose interests may be affected by the content of the manuscript. Disclosure represents a commitment to transparency and does not necessarily indicate a bias. If you are in doubt about whether to list a relationship/activity/interest, it is preferable that you do so.

The following questions apply to the author's relationships/activities/interests as they relate to the current manuscript only.

The author's relationships/activities/interests should be defined broadly. For example, if your manuscript pertains to the epidemiology of hypertension, you should declare all relationships with manufacturers of antihypertensive medication, even if that medication is not mentioned in the manuscript.

In item #1 below, report all support for the work reported in this manuscript without time limit. For all other items, the time frame for disclosure is the past 36 months.

		**Name all entities with whom you have this relationship or indicate none (add rows as needed)**	**Specifications/Comments (e.g., if payments were made to you or to your institution)**
**Time frame: Since the initial planning of the work**			
1	All support for the present manuscript (e.g., funding, provision of study materials, medical writing, article processing charges, etc.) **No time limit for this item.**	X None	
**Time frame: past 36 months**			
2	Grants or contracts from any entity (if not indicated in item #1 above).	X None	
3	Royalties or licenses	X None	

4	Consulting fees	X None	
5	Payment or honoraria for lectures, presentations, speakers bureaus, manuscript writing or educational events	____ None	Gilead, Fresenius Kabi, Biogen, Eli-Lilly, UCB, Abbvie, MSD, BMS
6	Payment for expert testimony	X None	
7	Support for attending meetings and/or travel	X None	
8	Patents planned, issued or pending	X None	
9	Participation on a Data Safety Monitoring Board or Advisory Board	X None	
10	Leadership or fiduciary role in other board, society, committee or advocacy group, paid or unpaid	X None	
11	Stock or stock options	X None	
12	Receipt of equipment, materials, drugs, medical writing, gifts or other services	X None	
13	Other financial or non-financial interests	X None	

Please place an "X" next to the following statement to indicate your agreement:

_X_ I certify that I have answered every question and have not altered the wording of any of the questions on this form.

ICMJE DISCLOSURE FORM

Date:____30 Oct 2025____
Your Name: Davide Gatti____
Manuscript Title: Romosozumab and Denosumab Combination Therapy After Denosumab in Postmenopausal Osteoporosis
Manuscript number (if known):____

In the interest of transparency, we ask you to disclose all relationships/activities/interests listed below that are related to the content of your manuscript. "Related" means any relation with for-profit or not-for-profit third parties whose interests may be affected by the content of the manuscript. Disclosure represents a commitment to transparency and does not necessarily indicate a bias. If you are in doubt about whether to list a relationship/activity/interest, it is preferable that you do so.

The following questions apply to the author's relationships/activities/interests as they relate to the current manuscript only.

The author's relationships/activities/interests should be defined broadly. For example, if your manuscript pertains to the epidemiology of hypertension, you should declare all relationships with manufacturers of antihypertensive medication, even if that medication is not mentioned in the manuscript.

In item #1 below, report all support for the work reported in this manuscript without time limit. For all other items, the time frame for disclosure is the past 36 months.

		**Name all entities with whom you have this relationship or indicate none (add rows as needed)**	**Specifications/Comments (e.g., if payments were made to you or to your institution)**
**Time frame: Since the initial planning of the work**			
1	All support for the present manuscript (e.g., funding, provision of study materials, medical writing, article processing charges, etc.) **No time limit for this item.**	X None	
**Time frame: past 36 months**			
2	Grants or contracts from any entity (if not indicated in item #1 above).	X None	
3	Royalties or licenses	X None	

4	Consulting fees	_X_ None	
5	Payment or honoraria for lectures, presentations, speakers bureaus, manuscript writing or educational events	____ None	Accord Health Care, Abiogen, Amgen, Eli-Lilly, Mereo,
			Neopharmed-Gentili, UCB
6	Payment for expert testimony	X__ None	
7	Support for attending meetings and/or travel	X__ None	
8	Patents planned, issued or pending	X__ None	
9	Participation on a Data Safety Monitoring Board or Advisory Board	_X_ None	
10	Leadership or fiduciary role in other board, society, committee or advocacy group, paid or unpaid	_X_ None	
11	Stock or stock options	_X_ None	
12	Receipt of equipment, materials, drugs, medical writing, gifts or other services	_X_ None	
13	Other financial or non-financial interests	__X None	

Please place an "X" next to the following statement to indicate your agreement:

_X_ I certify that I have answered every question and have not altered the wording of any of the questions on this form.

ICMJE DISCLOSURE FORM

Date:____30 Oct 2025________________________________
Your Name: Maurizio Rossini
Manuscript Title: Romosozumab and Denosumab Combination Therapy After Denosumab in Postmenopausal Osteoporosis
Manuscript number (if known):________________________________

In the interest of transparency, we ask you to disclose all relationships/activities/interests listed below that are related to the content of your manuscript. "Related" means any relation with for-profit or not-for-profit third parties whose interests may be affected by the content of the manuscript. Disclosure represents a commitment to transparency and does not necessarily indicate a bias. If you are in doubt about whether to list a relationship/activity/interest, it is preferable that you do so.

The following questions apply to the author's relationships/activities/interests as they relate to the current manuscript only.

The author's relationships/activities/interests should be defined broadly. For example, if your manuscript pertains to the epidemiology of hypertension, you should declare all relationships with manufacturers of antihypertensive medication, even if that medication is not mentioned in the manuscript.

In item #1 below, report all support for the work reported in this manuscript without time limit. For all other items, the time frame for disclosure is the past 36 months.

		**Name all entities with whom you have this relationship or indicate none (add rows as needed)**	**Specifications/Comments (e.g., if payments were made to you or to your institution)**
**Time frame: Since the initial planning of the work**			
1	All support for the present manuscript (e.g., funding, provision of study materials, medical writing, article processing charges, etc.) **No time limit for this item.**	X None	
**Time frame: past 36 months**			
2	Grants or contracts from any entity (if not indicated in item #1 above).	X None	
3	Royalties or licenses	X None	

4	Consulting fees	X None	
5	Payment or honoraria for lectures, presentations, speakers bureaus, manuscript writing or educational events	____ None	AbbVie, ItalFrarmaco, Neopharmed-Gentili, Eli-Lilly, Theramex, UCB
6	Payment for expert testimony	X None	
7	Support for attending meetings and/or travel	X None	
8	Patents planned, issued or pending	X None	
9	Participation on a Data Safety Monitoring Board or Advisory Board	X None	
10	Leadership or fiduciary role in other board, society, committee or advocacy group, paid or unpaid	X None	
11	Stock or stock options	X None	
12	Receipt of equipment, materials, drugs, medical writing, gifts or other services	X None	
13	Other financial or non-financial interests	X None	

Please place an "X" next to the following statement to indicate your agreement:

_X_ I certify that I have answered every question and have not altered the wording of any of the questions on this form.

ICMJE DISCLOSURE FORM

Date:____30 Oct 2025____
Your Name:__Francesco Pollastri____
Manuscript Title: Romosozumab and Denosumab Combination Therapy After Denosumab in Postmenopausal Osteoporosis
Manuscript number (if known):____

In the interest of transparency, we ask you to disclose all relationships/activities/interests listed below that are related to the content of your manuscript. "Related" means any relation with for-profit or not-for-profit third parties whose interests may be affected by the content of the manuscript. Disclosure represents a commitment to transparency and does not necessarily indicate a bias. If you are in doubt about whether to list a relationship/activity/interest, it is preferable that you do so.

The following questions apply to the author's relationships/activities/interests as they relate to the current manuscript only.

The author's relationships/activities/interests should be defined broadly. For example, if your manuscript pertains to the epidemiology of hypertension, you should declare all relationships with manufacturers of antihypertensive medication, even if that medication is not mentioned in the manuscript.

In item #1 below, report all support for the work reported in this manuscript without time limit. For all other items, the time frame for disclosure is the past 36 months.

		**Name all entities with whom you have this relationship or indicate none (add rows as needed)**	**Specifications/Comments (e.g., if payments were made to you or to your institution)**
**Time frame: Since the initial planning of the work**			
1	All support for the present manuscript (e.g., funding, provision of study materials, medical writing, article processing charges, etc.) **No time limit for this item.**	X None	
**Time frame: past 36 months**			
2	Grants or contracts from any entity (if not indicated in item #1 above).	X None	
3	Royalties or licenses	X None	

4	Consulting fees	_X_ None	
5	Payment or honoraria for lectures, presentations, speakers bureaus, manuscript writing or educational events	_X_ None	
6	Payment for expert testimony	_X_ None	
7	Support for attending meetings and/or travel	_X_ None	
8	Patents planned, issued or pending	_X_ None	
9	Participation on a Data Safety Monitoring Board or Advisory Board	_X_ None	
10	Leadership or fiduciary role in other board, society, committee or advocacy group, paid or unpaid	_X_ None	
11	Stock or stock options	_X_ None	
12	Receipt of equipment, materials, drugs, medical writing, gifts or other services	_X_ None	
13	Other financial or non-financial interests	_X_ None	

Please place an "X" next to the following statement to indicate your agreement:

_X_ I certify that I have answered every question and have not altered the wording of any of the questions on this form.
