# Supplementary material for: Romosozumab and Denosumab Combination Therapy After Denosumab in Postmenopausal Osteoporosis
Source: Arthritis Rheumatol. 2026 Feb 22;78(5):1176–83. doi: 10.1002/art.70002 (PMC13129633; doi:10.1002/art.70002)
Supplement: Supplementary file 2 — Appendix S1: Supplementary Information. [file ART-78-1176-s002.docx]

**Supplementary material**

**Romosozumab and Denosumab Combination Therapy After Denosumab in Postmenopausal Osteoporosis**

**Authors:** Giovanni Adami MD^1^, Francesco Pollastri MD^1^, Angelo Fassio MD^1^, Filippo Montanari MD^1^, Anna Piccinelli MD^1^, Camilla Benini MD^1^, Emma Pasetto MSc^1^, Carmen Dartizio MD^1^, Davide Gatti MD^1^, Maurizio Rossini MD^1^, Ombretta Viapiana MD^1^

1. Rheumatology Unit, University of Verona, Verona, Italy

**Table of Contents**

**Figure S1.** Study flowchart

**Study protocol**. Protocol for the prospective study

**Figure S1.** Study flowchart


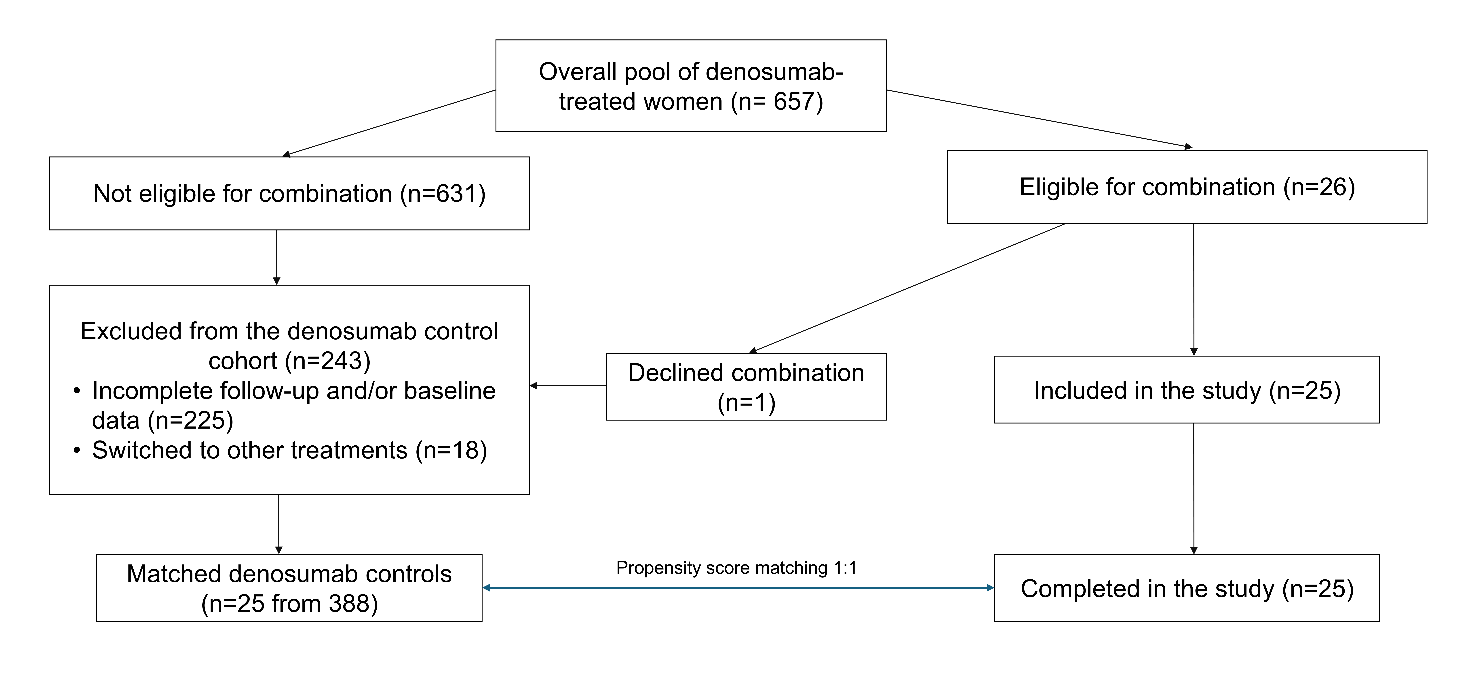


**Study protocol**

**Title:**

Prospective observational study on the effects of romosozumab added to ongoing denosumab in postmenopausal women with severe osteoporosis

Studio osservazionale sugli effetti della combinazione romosozumab-denosumab in pazienti con osteoporosi postmenopausale severa.

Version 1.0 - under the framework of the REUMABANK (Univ Verona) registry emend. 09/11/2022

Summary of changes

| **Protocol** | **Affected Section(s)** | **Summary of Revisions Made** | **Rationale** |
| --- | --- | --- | --- |
|  |  |  |  |
|  |  |  |  |
|  |  |  |  |
|  |  |  |  |
|  |  |  |  |
|  |  |  |  |

**Index**

[Title: 1](#_Toc207868440)

[Sponsor: 3](#_Toc207868441)

[Principal Investigator: 3](#_Toc207868442)

[1. Introduction and rationale 3](#_Toc207868443)

[2. Study design 3](#_Toc207868444)

[3. Study objectives 4](#_Toc207868445)

[4. Study population 4](#_Toc207868446)

[5. Study procedures 4](#_Toc207868447)

[6. Endpoints 5](#_Toc207868448)

[7. Data management and confidentiality 5](#_Toc207868449)

[8. Study duration 5](#_Toc207868450)

[9. Sample size considerations 5](#_Toc207868451)

[10. Statistical analysis 6](#_Toc207868452)

[11. Potential limitations and bias 6](#_Toc207868453)

[12. Ethical and privacy considerations 6](#_Toc207868454)

[13. Data and publication policy 7](#_Toc207868455)

[14. Benefit risk evaluation 7](#_Toc207868456)

[15. References 8](#_Toc207868457)

**Sponsor:**

Azienda Ospedalieria Universitaria Integrata, University Hospital of Verona – Rheumatology Unit - treatments and procedures will follow standard clinical practice

**Principal Investigator:**

Prof. Davide Gatti, Dr. Giovanni Adami

**1. Introduction and rationale**

Postmenopausal osteoporosis is one of the most prevalent chronic conditions affecting aging women, characterized by reduced bone mass, deterioration of bone microarchitecture, and a markedly increased risk of fragility fractures. Despite the availability of multiple pharmacologic options, a proportion of patients continues to sustain fractures, underscoring the need for improved treatment strategies in high-risk individuals.
Denosumab, a monoclonal antibody targeting RANKL, has become a cornerstone of therapy due to its potent antiresorptive action. However, it is not devoid of limitations. Patients may experience fractures while on long-term denosumab, and discontinuation of therapy is associated with a well-documented rebound phenomenon.
Romosozumab, an anti-sclerostin monoclonal antibody, exerts a dual mechanism of action: it stimulates bone formation while simultaneously reducing bone resorption. In treatment-naïve women, romosozumab has been shown to induce substantial BMD gains and reduce fracture risk. Preclinical studies and early clinical data suggest that romosozumab retains its anabolic capacity even when bone remodeling is suppressed.
Combining romosozumab with ongoing denosumab may therefore represent a rational therapeutic strategy. This protocol describes a prospective observational study focusing on postmenopausal women treated with denosumab for at least 24 months in whom romosozumab will be added. Retrospective data (M-24 to M0) may be retrieved to provide longitudinal context, but the central focus is the prospective observational phase (M0–M+12).

**2. Study design**

This is a prospective, single-centre, observational cohort study. Patients with severe postmenopausal osteoporosis currently treated with denosumab will be enrolled at the time of romosozumab initiation (M0). Follow-up visits will be conducted at 3, 6, and 12 months. Retrospective data may be collected, but only prospective data will be used for primary analyses.

**3. Study objectives**

Primary objective:

- To evaluate the effect of adding romosozumab to ongoing denosumab on lumbar spine BMD after 12 months.

Secondary objectives:

- To assess changes in femoral neck and total hip BMD
- To evaluate changes in bone turnover markers (P1NP, CTX)
- To describe the safety profile of the combination

**4. Study population**

Inclusion criteria:

- Postmenopausal women ≥50 years
- 10-year major osteoporotic fracture (MOF) risk ≥20%, assessed using the DeFRA
- T-score at the spine or femur of less than -2.5 (or less than -2.0 if there were ≥2 moderate or severe vertebral fractures or a femoral fracture in the previous two years)
- Signed informed consent

Exclusion criteria:

- MI or stroke history
- Other bone diseases
- Bone malignancy
- Severe liver/renal disease
- Uncontrolled endocrine disorders
- Prolonged bisphosphonate use
- Glucocorticoid or hormone-blocking therapy.

Sample size: 25 patients will be prospectively enrolled.

**5. Study procedures**

All enrolled patients will continue denosumab 60 mg/6mo and initiate romosozumab (210 mg monthly). Calcium and vitamin D supplementation will be provided.

All study visits and procedures will be performed in facilities University of Verona (Italy)

**Flowchart of visits and assessments:**

| Activity | Baseline (M0) | Month 3 | Month 6 | Month 12 |
| --- | --- | --- | --- | --- |
| Informed consent | **X** |  |  |  |
| Demographics & medical history | **X** |  |  |  |
| Concomitant medications | **X** | **X** | **X** | **X** |
| DXA (LS, FN, TH) | **X** |  | **X** | **X** |
| Blood samples (P1NP, CTX) | **X** | **X** | **X** | **X** |
| Safety assessments | **X** | **X** | **X** | **X** |

**6. Endpoints**

Primary endpoint:

- Percent change in lumbar spine BMD from baseline to month 12.

Secondary endpoints:

- Changes in femoral neck and total hip BMD
- Changes in P1NP and CTX
- Incidence of adverse events including CV events and fractures.

**7. Data management and confidentiality**

Data will be captured in REDCap, anonymized, GDPR-compliant. Access restricted to authorized staff. Paper documents stored securely for 7 years.

**8. Study duration**

Recruitment: 12 months; follow-up per patient: 12 months; total study duration: ~24 months.

**9. Sample size considerations**

A change in BMD of 6% in lumbar spine BMD corresponds to δ≈1.5. We would need a sample size of 11 in each group to reliably (with probability greater than or equal to 0.9) detect an effect size of |δ|≥1.5, assuming a two-sided criterion for detection that allows for a maximum Type I error rate of α=0.05.


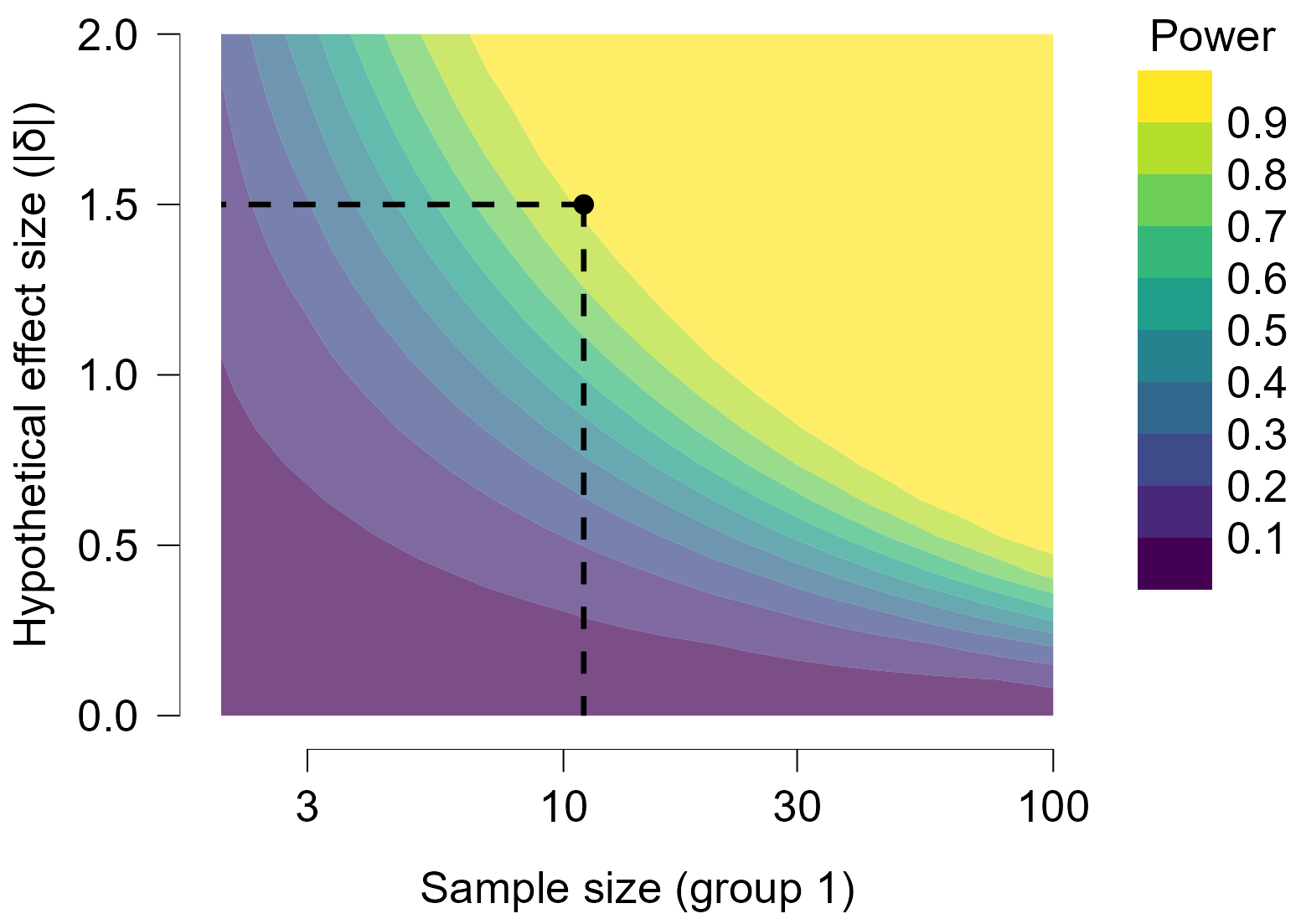


**10. Statistical analysis**

Baseline characteristics described with descriptive stats. Primary endpoint analyzed with MMRM including baseline BMD, time, patient as random effect. Secondary endpoints analyzed similarly. FDR adjustment for multiplicity. Interim analysis at 6 months will be provided.

**11. Potential limitations and bias**

As an observational study without randomization, results may be subject to residual confounding and selection bias. The sample size is limited, potentially underpowering the detection of modest treatment effects. Retrospective data may suffer from incompleteness or variability in timing. Fracture outcomes will not be systematically collected. Despite these limitations, the study provides essential exploratory evidence supporting future randomized trials.

**12. Ethical and privacy considerations**

Study conducted in accordance with the Declaration of Helsinki, ICH-GCP, and GDPR. Informed consent required. The Sponsor undertakes to comply with all applicable Privacy Laws (as defined herein), including those concerning security measures. The Sponsor guarantees, on its own behalf and on behalf of the Investigator, full awareness of all obligations arising from any applicable legislation regarding medical professional secrecy and the protection of patients’ personal data, including, but not limited to, Regulation (EU) 2016/679, the Italian Personal Data Protection Code (Legislative Decree no. 196/03, as subsequently amended), the measures, guidelines and current provisions issued by the Italian Data Protection Authority (collectively “Privacy Laws”). The Sponsor undertakes to ensure that all its personnel involved in the conduct of the study comply with the Privacy Laws and the Sponsor’s instructions regarding personal data protection, including aspects related to security and confidentiality of data. This obligation includes, by way of example but not limitation: (i) providing each study participant with a complete privacy notice in accordance with applicable law; (ii) obtaining the patient’s written informed consent prior to participation in the study (except for those patients for whom collection of consent is not possible); (iii) respecting the privacy rights of each subject as established by applicable Privacy Laws; (iv) adopting all appropriate technical and organizational security measures, in compliance with applicable Privacy Laws. The Sponsor will allow access to clinical data (including medical records) and any other information relevant to the study, in accordance with applicable Privacy Laws and while respecting both security measures and data confidentiality. For all remaining patients, prior to data collection, the Investigator or an authorized delegate undertakes to provide each patient with appropriate information, according to the model attached to this document, and to collect written informed consent from the patient: (a) to participate in the study; (b) to the processing of personal data.

**13. Data and publication policy**

Data owned by the sponsor (University Hospital of Verona). Results will be disseminated in peer-reviewed journals and conferences.

Data will be managed through the REDCap system of the University Hospital of Verona (https://redcap.aovr.veneto.it) and will be collected using an electronic Case Report Form (eCRF) created specifically for the study. REDCap (Research Electronic Data Capture) is a secure, web-based software platform designed to support data acquisition for research studies, providing: an intuitive interface for validated data entry; audit trails for tracking data manipulation and export procedures; automated export procedures for continuous download of data into common statistical packages; procedures for data integration and interoperability with external sources. The connection to the REDCap platform is encrypted using an SSL digital certificate, and database access is restricted to study personnel only, with two-factor authentication. REDCap provides a role-based user privilege system for data export, visualization, and modification. The University Hospital of Verona ensures daily backups. The platform generates a unique identifier code for each subject enrolled in the study, allowing investigators to maintain the association with identifying information locally. It should be noted, however, that when the data are used for scientific purposes, the possibility of identifying individual subjects will never be exercised, as this is neither useful nor necessary for the presentation of results. If a subject withdraws consent, no further data concerning that individual will be collected, while already collected data will continue to be used to determine research outcomes without modification. The Sponsor undertakes to retain original paper documentation (e.g., signed informed consent forms) for 7 years, in accordance with AIFA.

**14. Benefit risk evaluation**

The benefit-risk evaluation of both romosozumab and denosumab alone has been largely demonstrated by clinical trials and large observational studies. The combination of romosozumab and denosumab is expected to provide meaningful benefits for postmenopausal women with severe osteoporosis who remain at high fracture risk despite ongoing denosumab treatment. Romosozumab exerts a potent anabolic effect and has been shown, in pre-clinical studies, to significantly increase lumbar spine BMD even in the context of suppressed bone turnover, indicating that it retains anabolic capacity under denosumab therapy. By maintaining denosumab administration, the antiresorptive effect is preserved, thereby minimizing the risk of rebound-related bone loss or vertebral fractures.

The primary risks of the combination relate to the known safety profile of romosozumab, including the possible increase in cardiovascular events, and the general risks associated with long-term antiresorptive therapy. However, careful patient selection and ongoing monitoring will mitigate these risks. Importantly, the study excludes patients with a history of recent myocardial infarction or stroke, in line with current regulatory recommendations for romosozumab use. Hypocalcemia might be a possible risk, but it is unlikely to happen given the long-standing treatment with denosumab. Overall, the expected benefits in terms of skeletal outcomes outweigh the potential risks in this high-risk patient population. The observational design and close clinical monitoring will further ensure patient safety while providing valuable real-world evidence on the benefit–risk profile of this combination strategy.

**15. References**

1. Adami G, Fassio A, Gatti D, Viapiana O, Benini C, Danila MI, et al. Osteoporosis in 10 years time: a glimpse into the future of osteoporosis. Ther Adv Musculoskelet Dis 2022;14:1759720X221083541.

2. Ramchand SK, Leder BZ. Sequential Therapy for the Long-Term Treatment of Postmenopausal Osteoporosis. The Journal of Clinical Endocrinology & Metabolism 2024;109:303–11.

3. Cummings SR, San Martin J, McClung MR, Siris ES, Eastell R, Reid IR, et al. Denosumab for prevention of fractures in postmenopausal women with osteoporosis. N. Engl. J. Med. 2009;361:756–65.

4. Anastasilakis AD, Polyzos SA, Makras P, Aubry-Rozier B, Kaouri S, Lamy O. Clinical Features of 24 Patients With Rebound-Associated Vertebral Fractures After Denosumab Discontinuation: Systematic Review and Additional Cases. J. Bone Miner. Res. 2017;32:1291–6.

5. Kim AS, Girgis CM, McDonald MM. Osteoclast Recycling and the Rebound Phenomenon Following Denosumab Discontinuation. Curr Osteoporos Rep 2022;20:505–15.

6. Saag KG, Petersen J, Brandi ML, Karaplis AC, Lorentzon M, Thomas T, et al. Romosozumab or Alendronate for Fracture Prevention in Women with Osteoporosis. N. Engl. J. Med. 2017;377:1417–27.
